# Association between coat colour and the behaviour of Australian Labrador retrievers

**DOI:** 10.1186/s40575-019-0078-z

**Published:** 2019-11-30

**Authors:** Diane van Rooy, Claire M. Wade

**Affiliations:** The University of Sydney, Camperdown, NSW 2006 Australia

**Keywords:** Labrador, Behaviour, Colour, MC1R, TYRP1

## Abstract

**Background:**

Making assumptions regarding temperament and intelligence based on the physical appearance of dogs can be a conscious or unconscious human act. Labrador retrievers with chocolate-coloured coats are anecdotally considered to be less trainable and more hyperactive and aggressive than their black or yellow peers. To test these assertions, we analysed the owner-reported behavioural traits of Labradors in relation to both their observable coat colour, and their *TYRP1* and *MC1R* genotypes.

**Results:**

We used the results of an owner-based questionnaire to determine scores for 21 behavioural traits and test whether these scores varied with coat colour (*n* = 225). *Familiar dog aggression* was the only trait that was found to vary significantly with coat colour (*P* = 0.013). Yellow Labradors had a higher score than chocolate Labradors, even when corrected for multiple testing (*P* = 0.021).

We repeated the analyses for a subset of 63 Labradors with available genotyping data for the genes (MC1R and TYRP1) that are known to determine the primary coat colours in Labradors. *Familiar dog aggression* scores varied with both the observed coat colour and *MC1R* genotype. Dogs homozygous for *MC1R* recessive allele (with yellow coat colour) scored higher for *familiar dog aggression* than either black or chocolate Labradors. However, no association maintained significance when incorporating Bonferroni correction. Dog *trainability* scores decreased additively as the number of recessive brown alleles for *TYRP1* increased. This allelic association was independent of the observable coat colour. Dogs homozygous for the brown allele were considered less trainable than dogs with no brown alleles (*P* = 0.030).

**Conclusions:**

Our results do not support that chocolate-coloured Labradors are more hyperactive or aggressive than either black or yellow Labradors. Trainability scores varied with *TYRP1* genotype but not the observable coat colour. Further validation is required.

## Plain English summary

Anecdotally, chocolate-coloured Labrador retrievers have a reputation for being harder to train and more hyperactive and aggressive than yellow or black Labradors. This may be due to preconceived bias. To date, there is little scientific data to support these beliefs. To put it to the test, we used chocolate, yellow or black Labradors exhibiting a range of behavioural characteristics as reported in an owner-based questionnaire.

We considered 21 behavioural traits in 225 Labrador retrievers. Only *familiar dog aggression* varied with coat colour, with yellow Labradors having a significantly higher score than chocolate Labradors. We then analysed a subgroup of 63 Labradors with additional genotyping data for the two main genes involved in Labrador coat colour. *Trainability* was significantly lower in dogs with two *b* (brown) alleles of *TYRP1* compared to dogs with no brown alleles.

Our results do not support the suggestion that chocolate-coloured Labradors are more hyperactive or aggressive than their yellow or black peers. Chocolate Labradors actually showed less aggression to familiar dogs than yellow Labradors. However, dog trainability declined as the number of copies of the recessive allele responsible for the chocolate coat colour increased. Further validation with an increased sample size is required.

## Background

When Labrador retrievers were first recognized by the national Kennel Clubs in England in 1903 and the USA in 1917, black was the predominant coat colour. The first appearance of chocolate Labradors can be traced back to a litter born in 1892 and sired by Buccleuch Avon: the first yellow Labrador on record is Ben of Hyde, born in 1899 [1, 2]. While there has been a rapid rise in the popularity of yellow Labradors, fans of Labrador retrievers may have noticed that chocolate Labradors are less common than black or yellow Labradors. Anecdotally, chocolate-coloured Labradors have a reputation for being less trainable and more hyperactive and aggressive than their black or yellow peers. To date there is little scientific evidence to support this belief.

It is well known that people can have preconceived ideas about the personalities of dogs based on their appearance. For example, earlier work has shown that dogs perceived as being cute are more likely to be perceived as amicable [3]. Based on appearance alone, dogs with yellow coat colour are assessed as being more agreeable, conscientious and emotionally stable than dogs that are otherwise identical other than having a black coat [4]. Similarly, dogs with floppy ears are considered to be more agreeable and emotionally stable than dogs with pointy ears [4].

Coat colour is determined by melanocytes producing either phaeomelanin resulting in a yellow or red coat; or eumelanin resulting in a brown or black coat. The three recognized coat colours in Labrador retrievers are black, chocolate and yellow. Observable within-breed variation among coat colours is determined by two genes: *MC1R* (melanocortin 1 receptor) and *TYRP1* (tyrosinase related protein 1). Black is the dominant colour at the *TYRP1* locus while brown coat colour is recessive. Yellow Labradors are homozygous for a recessive *MC1R* mutation (R306ter), a nucleotide alteration that causes a premature stop codon at amino acid 306 of *MC1R*, resulting in blocking of eumelanin production allowing only the phaeomelanin reds and yellows to show [5–7]. This is also known as the *E* locus. Labradors with the recessive *bb* genotype at the B locus of *TYRP1* can be chocolate or yellow, while those with genotypes *BB* or *Bb* can be black or yellow. Several mutations at the brown locus may cause the brown phenotype [5].

Yellow has recently overtaken black as the most popular colour in registered Labradors in the UK (Kennel Club registration data). The popularity of chocolate Labradors has consistently been far lower than black or yellow, but does vary: 7% in 1988, 22% in 2008 and 9% in 2018 (Kennel Club registration data). Unfortunately similar data for Australian or American Labradors was not available, preventing regional comparisons. A UK study found that chocolate Labradors (21%) weighed, on average, 1.4 kg more than black (49%) and yellow Labradors (27%) [8]. Recent work suggested that, of 2074 Labradors with health records, chocolate Labradors were more likely to have otitis externa and pyo-traumatic dermatitis than either black or yellow Labradors. Data on longevity for 173 Labradors revealed that the median lifespan for chocolate Labradors was significantly less than for non-chocolate Labradors (10.7 years compared to 12.1 years) [9]. Whether the increased propensity for skin or ear infections is related to longevity is yet to be determined. It is also currently unclear whether the same differences are characteristic of Labradors in other countries.

The mechanism by which coat colour may affect behaviour is yet to be definitively determined, but various hypotheses abound. Melanocortins such as adrenococorticotropic hormone (ACTH) and melanocyte stimulating hormone (MSH) bind to the melanocortin 1 receptors in the skin responsible for coat colour, but also bind to other melanocortin receptors [10]. *MC2R* mediates the effect of ACTH on steroid secretion in the adrenal gland. *MC3R* and *MC4R* are expressed in the brain, especially the hypothalamus. *MC5R* has been linked to aggressive behaviour in mice [10]. Consequently, melanocortins may be involved in many behavioural and physiological functions. Lines of mice carrying *TYRP1* mutations have also exhibited behavioural abnormalities. For instance, Tyrp1^b-1FGHLc^/Tyrp1^b-1FCHLc^ strains of mice exhibit brown pigmented eumelanin, decreased eye pigment, decreased body size, and are described as nervous. (http://www.informatics.jax.org/allele/genoview/MGI:3719250?counter=1).

The goal of this study was to test claims regarding chocolate Labrador retrievers and temperament differences relative to other coat colours. To do this, we used dogs that have results available for both array-based genotyping data and behaviour characteristics assessed by their owners using a standardized questionnaire. By using the allelic haplotypes at the two relevant genes rather than simply the observed phenotypic coat colour, we were able to observe the association of allele dosage at each locus, and the relative impacts of the different genetic backgrounds on behaviour.

## Results

Questionnaire data were available for 92 black, 99 yellow, and 34 chocolate Labradors. Demographic information for these dogs can be viewed in Additional file 1: Table S1. While dogs were not required to be registered with the ANKC, 143 dogs were reported to be acquired from breeders. An MDS plot of the 63 genotyped dogs was consistent with all being Labrador retrievers.

**Fig. 1:**
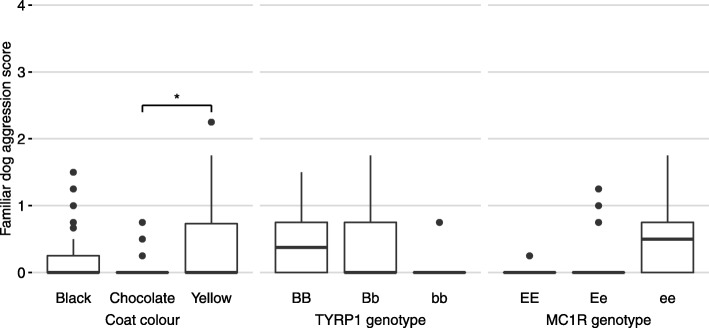
boxplots of Familiar dog aggression score. Significant differences between the groups calculated by Mann Whitney U test with Bonferroni correction. Coat colour (*n* = 195), TYRP1 genotype (*n* = 54), MC1R genotype (*n* = 54) *: *P* < 0.05

For each of 21 behavioural traits listed in Table 1, dogs of different coat colours were compared. When black and yellow Labradors were combined and compared to chocolate Labradors, there was no significant difference in any of the traits. When black, yellow and chocolate were considered separately, the only trait which varied significantly with coat colour was *familiar dog aggression* which is also referred to as *dog rivalry* in earlier versions of C-BARQ (*P* = 0.013). Yellow Labradors demonstrated a higher score for *familiar dog aggression* relative to black (*P* = 0.037) and chocolate coat colours (*P* = 0.007). After correction for multiple testing, the difference between yellow and chocolate Labradors remained significant (*P* = 0.021) (Fig. 1).

**Table 1:** Comparing scores for 21 behavioural traits in 225 Labrador retrievers based on coat colour

Behaviour Trait	*P*
Agitated when ignored	0.150
Attachment/Attention-seeking behaviour	0.064
Barking	0.627
Chasing behaviour	0.550
Coprophagia	0.106
Dog-directed aggression	0.323
Dog-directed fear	0.336
Energy levels	0.518
Excitability	0.798
Familiar dog aggression	**0.013**
Licking behaviour	0.311
Mounting behaviour	0.866
Noise fear	0.408
Non-social fear	0.130
Owner-directed aggression	0.224
Separation-related behaviour	0.608
Stranger-directed aggression	0.961
Stranger-directed fear	0.062
Touch sensitivity	0.543
Trainability	0.737
Unusual behaviours	0.666

*P*: chi-square probability using Kruskal-Wallis nonparametric test to compare black, yellow and chocolate Labradors

Probability in bold typeface indicates *P* <0.05

*P*: chi-square probability using Kruskal-Wallis nonparametric test to compare black, yellow and chocolate Labradors.

Probability in bold typeface indicates *P* < 0.05.

The scores for *familiar dog aggression* still varied significantly with coat colour when possible confounding demographic factors were taken into account (*P* = 0.010). Stepwise regression resulted in coat colour and the number of dogs in the household being the only demographic factors retained in a reduced model.

Of the dogs with questionnaire data, 63 Labradors had genotyping data (Table 2). Each behavioural trait was examined according to scores for dogs grouped by coat colour, *TYRP1* genotype and *MC1R* genotype (Table 3). Only one Labrador was homozygous recessive at both loci, limiting our power to assess interaction effects. All dogs had external phenotypes that were concordant with expectation given the phenotype.

**Table 2:** Colour genotypes of 63 Labrador retrievers

	Black	Yellow	Chocolate
*n*	29	24	10
*MC1R* genotype			
EE	5	0	3
Ee	24	0	7
ee	0	24	0
*TYRP1* genotype			
BB	7	4	0
Bb	22	19	0
bb	0	1	10

**Table 3:** Comparing scores for 21 behavioural traits in 63 genotyped Labrador retrievers using Kruskal-Wallis nonparametric test

Behaviour Trait	*P -* colour	*P - TYRP1*	*P- MC1R*
Agitated when ignored	0.657	0.961	0.535
Attachment/Attention-seeking behaviour	0.673	0.347	0.331
Barking	0.800	0.843	0.825
Chasing behaviour	0.416	0.528	0.797
Coprophagia	0.100	0.344	0.054
Dog-directed aggression	0.922	0.954	0.990
Dog-directed fear	0.373	0.186	0.740
Energy levels	0.667	0.527	0.763
Excitability	0.998	0.532	0.742
Familiar dog aggression	**0.040**	0.130	**0.029**
Licking behaviour	0.486	0.747	0.620
Mounting behaviour	0.683	0.783	0.858
Noise fear	0.397	0.276	0.892
Non-social fear	0.302	0.172	0.913
Owner-directed aggression	0.183	0.406	0.243
Separation-related behaviour	0.981	0.935	0.857
Stranger-directed aggression	0.698	0.542	0.515
Stranger-directed fear	0.249	0.133	0.243
Touch sensitivity	0.545	0.730	0.497
Trainability	0.527	**0.033**	0.787
Unusual behaviours	0.328	0.544	0.431

*P* - colour: chi-square probability comparing black, yellow and chocolate Labradors

*P - TYRP1* genotype: chi-square probability comparing BB, Bb and bb genotype

*P - MC1R*: genotype: chi-square probability comparing EE, Ee and ee genotype

Probability in bold typeface indicates *P* <0.05

Once more, *familiar dog aggression* demonstrated differences according to coat colour as well as *MC1R* genotype. Homozygous *MC1R* variant Labradors demonstrated a higher score than both dogs homozygous for the dominant *E* allele (*P* = 0.024) and heterozygotes (*P* = 0.034). However this did not maintain significance when corrected for multiple testing.

**Fig. 2:**
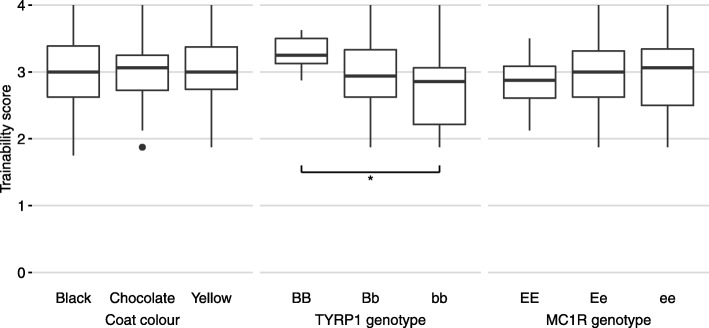
boxplots of Trainability score. Significant differences between the groups calculated by Mann Whitney U test with Bonferroni correction. Coat colour (*n* = 224), TYRP1 genotype (*n* = 63), MC1R genotype (*n* = 63) *: *P* < 0.05

*Trainability* varied significantly according to *TYRP1* genotype but not observable coat colour. Dogs with *bb* genotype scored lower than both heterozygous dogs (*P* = 0.415) and dogs with BB genotype (*P* = 0.010), while heterozygous dogs scored lower than dogs with BB genotype (*P* = 0.028). (Fig. 2). After Bonferroni correction, the difference between dogs with BB and bb genotypes retained significance (*P* = 0.030).

Stepwise regression removed all variables for both *familiar dog aggression* and *trainability* in the genotyped subgroup, leaving no demographic factors in the reduced models.

*P* - colour: chi-square probability comparing black, yellow and chocolate Labradors.

*P* - *TYRP1* genotype: chi-square probability comparing BB, Bb and bb genotype.

*P* - *MC1R*: genotype: chi-square probability comparing EE, Ee and ee genotype.

Probability in bold typeface indicates *P* < 0.05.

## Discussion and conclusion

The potential relationship between coat colour and behaviour has previously been explored in a study of personality traits in Labrador retrievers in the UK [11, 12]. Analysis of C-BARQ data gathered on 1144 black Labradors, 521 yellow Labradors and 310 chocolate Labradors demonstrated a statistically significant association between coat colour and nine of twelve personality traits assessed in the study. For example, chocolate Labradors were scored as becoming more agitated when ignored by their human guardians than black Labradors and were more excitable than black Labradors. Chocolate Labradors were regarded by their owners as less trainable than either black or yellow Labradors. As a positive difference, chocolate Labradors showed less fear of noises than either black or yellow Labradors. In comparison, we found no difference in scores for *agitated when ignored, noise fear* or *excitability* between black, yellow or chocolate Labradors. Our sample was much smaller, containing only 34 chocolate Labradors with questionnaire data, and 10 chocolate Labradors with both questionnaire and genotyping data. There was also a difference in the scoring of the questionnaires. C-BARQ scores aggression, anxiety and excitability traits based on severity, while other traits are based on frequency. Our questionnaire, while largely modelled on C-BARQ did differ in that all traits were scored according to frequency of behaviours. It is also possible that differences in the reported behaviours of Labradors may be affected by their geographic region, or may be affected by the purposes for which they are bred [13]. The within-breed genetic variation of Labradors in the past has been associated with both the role of the dog (working, show, pet) and coat colour [14]. Chocolate Labradors were primarily located within the cluster of show dogs while the black and yellow Labradors were more likely to cluster with the gundogs [14]. This supports the anecdotal view in the UK that chocolate Labradors are considered to be more successful in the show ring than in field trials. Our participants were mainly companion dogs.

However, our findings did align with those of some previous studies. In a UK study, undesired behaviours (not defined) were reported in 3.3% of black Labradors, 2.1% of yellow Labradors but only 1.8% of chocolate Labradors [9]. Black Labradors took longer to learn a reversal learning task than yellow Labradors and committed more errors [15]. Of 28 black, 20 yellow and 8 chocolate Labrador retrievers living in Australian backyards, yellow Labradors were observed to exhibit an increased likelihood of problem behaviours (barking, digging, object manipulation, chewing objects) (r = 0.3, *P* < 0.01) compared with Labradors of other colours [16]. Lack of training was linked to increased problem behaviours only in the yellow Labradors (*P* < 0.001). However, exposure to training and the inherent trainability of the dog are not the same. At the Cornell University Animal Behaviour clinic, reported colours of Labradors seen at the clinic for aggression were compared with the relative proportions of colours of Labradors presented to their Veterinary Medicine Teaching Hospital for other reasons [17]. Although chocolate Labradors comprised 18% of the caseload of Labradors seen at the hospital, only 7% of the Labradors being assessed at the behaviour clinic for aggression were chocolate. By contrast, black Labradors presented for aggression at the predicted rate (52% for both), while yellow Labradors were over-represented for aggression cases, making up 30% of those seen in the general practice clinic but 41% of those that presented at the behaviour clinic for aggression.

Our study found no evidence that chocolate Labradors varied significantly in their behaviours in comparison with non-chocolate Labradors in our larger cohort, tested by grouping black and yellow Labradors. We found no evidence that chocolate-coloured Labradors are more hyperactive or aggressive than black or yellow Labradors. In fact, the yellow Labradors had a higher score for *familiar dog aggression*.

Dog *trainability* was associated with *TYRP1* genotype but was related to the allele dosage rather than the colour per se, since black dogs heterozygous for the brown allele had reduced trainability. Research by aficionados of the breed has detected that the brown alleles may have been introduced by interbreeding with other breeds such as the Chesapeake Bay retriever and the flat-coated retriever [1]. The introgressed genes from other breeds may have impacted behaviour more broadly, as the working characteristics of the sporting breeds vary considerably according to original purposes of the breeds. Nonetheless, it is interesting that statistical significance tracks directly with the allelic dosage at the brown locus.

## Materials and methods

### Participants and questionnaire

This was an opportunistic analysis of questionnaire data from Australian Labrador retrievers recruited for a study on separation-related distress between May 2010 and May 2016. Participating owners and dogs were recruited by answering advertisements on dog-related websites, in magazines, at veterinary clinics, obedience clubs and boarding kennels, or by word of mouth. Both affected and control dogs were targeted in the recruitment, and all participants were self-selected. The Australian Dog Behaviour Survey has been described in published work on this study [18, 19] and is available in Additional file 3. The questionnaire was based largely on the validated Canine Behaviour and Research Questionnaire (C-BARQ) [20]. The main difference was that frequency was used to assess all traits. Scores for behaviour traits were calculated as per Additional file 2: Table S2. All questions had an option of answering “Not applicable or observed”; if selected these were treated as missing values and were ignored.

In the questionnaire, breed and coat colour were reported by owners. Source of acquisition included *Breeder* but proof of ANKC registration was not required. Owners were given the options of describing their dog as being of black, yellow or chocolate coat colour.

### Genetic analysis

A proportion of the dogs (63 of 225) with questionnaire data had been genotyped using Illumina CanineHD Whole-Genome genotyping beadchip arrays containing either 170,000 or 220,000 markers (Neogen/Geneseek, Nebraska USA). Markers at 11.33326416, 11.33337736, 11.33347564 and 5.63683288,5.63694982, 5.63697949, 5.63710280,5.63718071, 5.63728735 enabled the dogs’ genotypes to be imputed for *TYRP1* and *MC1R* loci respectively.

### Statistical analysis

For each of the 21 behaviour traits, the Kruskal Wallis test was used to determine if there were statistical differences between dogs of different coat colours. In the smaller subset of genotyped dogs, the same test compared groups based on coat colour, imputed TYRP1 genotype and imputed MC1R genotype. Where there was a statistically significant difference between group (*P* < 0.05), further testing with Mann Whitney U test was carried out. Results were then subjected to Bonferroni correction. Nonparametric tests were employed as the data was not normally distributed.

Regression analysis using a Poisson distribution and Logarithm link function was applied only to those traits showing significant results for the above tests. Covariates included age at survey, age acquired, sex/reproductive status, source of acquisition, number of dogs in household as well as either coat colour or genotype.

## Supplementary information

**Additional file 1:** **Table S1.** Demographic information on participating dogs. (DOCX 26 kb)

**Additional file 2: Table S2.** Australian Canine Behaviour Survey behavioural traits, calculating scores and their definitions. (DOCX 16 kb)

**Additional file 3:** Australian Canine Behaviour Survey.

## Data Availability

Datasets detailing participants are available in the supplementary information files. Additional datasets used and/or analysed during this study are available from the corresponding author on reasonable request.
