# Supplementary material for: Association between coat colour and the behaviour of Australian Labrador retrievers
Source: Canine Genet Epidemiol. 2019 Nov 30;6:10. doi: 10.1186/s40575-019-0078-z (PMC6884874; doi:10.1186/s40575-019-0078-z)
Supplement: Supplementary file 1 — Additional file 1: Table S1. Demographic information on participating dogs. (DOCX 26 kb) [file 40575_2019_78_MOESM1_ESM.docx]

Table S1: Demographic information on participating dogs

| **Coat colour** | **Age (years)** | **Age acquired** | **Sex/Reprod. Status** | **Source of acquisition** | **No. dogs in household** | **Genotyped** |
| --- | --- | --- | --- | --- | --- | --- |
| Yellow | 3.5 | 2-3 months | MN | Pet Shop | 2 | Yes |
| Yellow | 6.9 | 6-12 months | FN |  | 2 |  |
| Yellow | 13.2 | 2-3 months | MN | Breeder | 2 | Yes |
| Yellow | 9.2 | 2-3 months | MN |  | 1 | Yes |
| Black | 13.1 | <2 months | FN | Breeder | 3 |  |
| Black | 1.4 | <2 months | FN | Breeder | 3 | Yes |
| Black | 7.3 | <2 months | FN | Breeder | 3 |  |
| Yellow | 5.7 | <2 months | MN | Breeder | 1 | Yes |
| Yellow | 4.7 | 1-2 years | FN | Assistance Dogs | 2 |  |
| Yellow | 7.7 | 2-3 months | MN | Breeder | 1 | Yes |
| Yellow | 5.1 | 2-3 months | MN | Breeder | 1 | Yes |
| Black | 11.4 | <2 months | M | Breeder | 4+ | Yes |
| Black |  | <2 months | F | Breeder | 4+ | Yes |
| Black |  | <2 months | F | Breeder | 4+ |  |
| Yellow | 3 | <2 months | F | Breeder | 4+ |  |
| Black | 1.5 | <2 months | F | Breeder | 4+ |  |
| Yellow | 1.3 | <2 months | F | Breeder | 4+ | Yes |
| Yellow | 8.3 | 2-6 years | FN | Breeder | 4+ |  |
| Black | 11.5 | 1-2 years | FN | Friend | 2 |  |
| Yellow |  | 2-3 months | MN | Breeder | 1 |  |
| Yellow | 10.2 | 2-3 months | M | Breeder | 1 | Yes |
| Yellow | 3.1 | 2-3 months | FN | Breeder | 1 |  |
| Black | 5.4 | 2-6 years | M | Friend | 1 | Yes |
| Chocolate | 2.1 | <2 months | F | Breeder | 4+ | Yes |
| Yellow | 4.3 | <2 months | MN | Breeder | 4+ | Yes |
| Yellow | 3.3 | 2-3 months | FN | Breeder | 1 |  |
| Yellow | 5.4 | <2 months | MN | Breeder | 4+ | Yes |
| Black | 8.1 | 2-3 months | FN | Breeder | 4+ |  |
| Chocolate | 1.6 | 2-3 months | FN | Pet Shop | 2 |  |
| Yellow | 11.4 | 1-2 years | MN | Friend | 2 |  |
| Yellow | 8.7 | >6 years | FN | Shelter | 1 |  |
| Black | 4.7 | <2 months | FN | Breeder | 1 | Yes |
| Yellow | 3.6 | 3-6 months | FN | Friend | 2 |  |
| Black | 10.4 | <2 months | MN | Breeder | 2 |  |
| Yellow | 12.2 | 2-3 months | FN | Breeder | 2 |  |
| Black | 6.2 | 2-3 months | FN | Breeder | 3 | Yes |
| Black | 7.9 | <2 months | MN | Breeder | 2 | Yes |
| Yellow | 5.5 | 2-3 months | MN | Breeder | 1 |  |
| Chocolate | 7.7 | 2-6 years | FN | Breeder | 2 | Yes |
| Chocolate | 7.7 | 2-6 years | FN | Breeder | 2 | Yes |
| Yellow | 1.0 | 3-6 months | MN | Breeder | 1 |  |
| Black | 6.6 | >6 years | MN | Assistance Dogs | 2 |  |
| Yellow | 4.8 | <2 months | MN | Friend | 2 |  |
| Chocolate | 2.9 | <2 months | MN | Assistance Dogs | 2 |  |
| Yellow | 1.2 | <2 months | FN |  | 1 |  |
| Yellow | 2.4 | 6-12 months | FN | Friend | 1 |  |
| Yellow | 11.9 | 1-2 years | MN | Assistance Dogs | 1 |  |
| Yellow | 3.0 | 2-3 months | MN | Breeder | 2 |  |
| Yellow | 7.5 | 2-3 months | FN | Breeder | 2 | Yes |
| Black | 0.8 | 2-3 months | M |  | 1 |  |
| Black | 1 | 3-6 months | MN | Breeder | 1 |  |
| Black | 4.0 | 3-6 months | MN | Breeder | 1 |  |
| Chocolate | 5.4 | 2-6 years | MN | Assistance Dogs | 2 |  |
| Chocolate | 3.1 | 1-2 years | FN | Assistance Dogs | 2 |  |
| Chocolate | 3.6 | <2 months | FN | Breeder |  |  |
| Black | 4.4 | 1-2 years | FN | Friend | 2 |  |
| Yellow | 2.7 | 1-2 years | MN | Friend | 3 |  |
| Yellow | 3.8 | 3-6 months | FN | Breeder | 3 | Yes |
| Chocolate | 1.4 | 2-3 months | MN | Breeder | 1 | Yes |
| Yellow |  | 6-12 months | FN | Shelter | 2 |  |
| Black | 3.3 | <2 months | F | Breeder | 4+ |  |
| Chocolate | 5.3 | 3-6 months | MN | Breeder | 2 | Yes |
| Chocolate | 2.2 | <2 months | FN | Breeder | 1 |  |
| Black | 9.6 | 2-3 months | MN | Breeder | 2 |  |
| Chocolate | 6.6 | 2-3 months | FN | Breeder | 2 |  |
| Black | 3.0 | <2 months | F |  |  |  |
| Yellow | 9.2 | 2-3 months | FN | Breeder |  |  |
| Chocolate | 3.6 | <2 months | MN | Breeder |  |  |
| Black | 5.4 | 6-12 months | M | Breeder | 3 |  |
| Black | 10.6 | <2 months | F | Breeder | 2 | Yes |
| Yellow | 7.1 | <2 months | MN | Breeder | 3 |  |
| Yellow | 4.1 | 2-3 months | MN | Assistance Dogs | 2 |  |
| Yellow | 7.5 | 3-6 months | FN | Pet Shop | 1 |  |
| Chocolate | 5.5 | 3-6 months | F | Breeder | 1 |  |
| Black | 3.7 | 2-3 months | FN | Breeder | 1 |  |
| Black | 1.7 | 1-2 years | FN | Shelter | 2 | Yes |
| Black | 1.4 | <2 months | FN | Breeder | 2 |  |
| Yellow | 6.7 | <2 months | MN | Breeder | 1 |  |
| Yellow | 10.1 | 2-3 months | FN | Breeder | 2 | Yes |
| Yellow | 2.5 | <2 months | FN | Breeder | 1 | Yes |
| Chocolate | 2.5 | 2-3 months | FN | Breeder | 4+ |  |
| Black | 5.6 | 2-3 months | FN | Assistance Dogs | 2 |  |
| Black | 5.8 | <2 months | FN | Breeder | 3 |  |
| Black | 15.0 | 2-3 months | FN | Friend | 2 |  |
| Yellow | 3.7 | 2-3 months | F | Breeder | 1 |  |
| Yellow | 4.5 | <2 months | MN | Shelter | 1 | Yes |
| Black | 2.4 | 2-3 months | FN | Breeder | 1 |  |
| Chocolate | 3.5 | 2-3 months | MN | Breeder | 2 | Yes |
| Black | 5.4 | 1-2 years | FN | Friend | 1 | Yes |
| Chocolate | 1.4 | <2 months | MN | Friend | 1 |  |
| Black | 5.5 | <2 months | FN | Breeder | 3 | Yes |
| Black | 5.5 | <2 months | FN | Pet Shop | 3 | Yes |
| Yellow | 1.9 | 3-6 months | F | Breeder | 1 |  |
| Black | 8.6 | 2-3 months | FN | Breeder | 2 |  |
| Black | 4.2 | <2 months | F | Breeder | 4+ |  |
| Yellow | 9.5 | 2-3 months | FN | Pet Shop | 1 |  |
| Black | 4.4 | 1-2 years | FN | Assistance Dogs | 1 |  |
| Chocolate | 4.6 | 2-3 months | FN | Pet Shop | 1 | Yes |
| Yellow | 8.0 | 2-3 months | MN | Breeder | 2 | Yes |
| Yellow | 2.4 | <2 months | F | Breeder | 2 |  |
| Chocolate | 3.1 | 3-6 months | FN |  | 1 | Yes |
| Yellow | 2.6 | <2 months | F | Breeder | 1 |  |
| Black | 1.8 | 2-3 months | MN | Breeder | 1 |  |
| Black | 2.5 | 6-12 months | F | Breeder | 2 |  |
| Black | 1.0 | 2-3 months | F | Breeder | 2 |  |
| Yellow | 10.1 | 2-3 months | FN | Breeder | 4+ |  |
| Black | 11.1 | <2 months | M | Breeder | 4+ |  |
| Yellow | 3.3 | 1-2 years | MN | Shelter | 2 |  |
| Black | 9.7 | 2-3 months | MN | Breeder | 2 |  |
| Black | 5.8 | 2-3 months | FN | Pet Shop | 2 | Yes |
| Yellow | 4.4 | 2-3 months | MN | Pet Shop | 2 |  |
| Chocolate | 6.3 | <2 months | FN | Breeder | 1 | Yes |
| Yellow | 11.5 | 3-6 months | FN | Shelter | 1 |  |
| Black | 5.0 | 1-2 years | FN | Breeder | 1 |  |
| Yellow | 4.4 | <2 months | FN | Breeder | 1 | Yes |
| Yellow | 4.2 | <2 months | MN | Pet Shop | 1 |  |
| Yellow | 9.1 | <2 months | FN | Breeder | 3 |  |
| Black | 2 | <2 months | FN | Breeder | 2 | Yes |
| Black | 1.8 | 2-3 months | M | Breeder | 2 |  |
| Yellow | 3.7 | 2-3 months | MN | Breeder | 3 |  |
| Yellow | 5.9 | 2-3 months | MN | Breeder | 2 | Yes |
| Yellow | 7 | 2-6 years | FN | Shelter | 1 |  |
| Black | 8.6 | <2 months | FN | Breeder | 1 |  |
| Yellow | 2.7 | 2-3 months | FN | Breeder | 1 |  |
| Black | 7.1 | 1-2 years | MN |  | 1 |  |
| Black | 1.7 | 2-3 months | MN | Assistance Dogs | 2 |  |
| Yellow | 1.1 | 2-3 months | MN | Assistance Dogs | 1 |  |
| Yellow | 4.4 | <2 months | MN | Breeder | 1 | Yes |
| Black |  | 2-6 years | MN | Shelter | 1 |  |
| Yellow | 4.1 | 2-3 months | MN | Breeder | 2 | Yes |
| Black | 2.8 | 2-3 months | FN | Breeder | 2 | Yes |
| Black | 10.8 | 2-3 months | FN | Friend | 2 |  |
| Black | 9.0 | <2 months | FN | Friend | 2 |  |
| Black | 1.5 | 3-6 months | FN | Breeder | 1 |  |
| Black | 2.1 | 2-3 months | FN | Assistance Dogs | 1 |  |
| Yellow | 5.7 | 6-12 months | FN | Breeder | 3 |  |
| Black | 7.1 | 2-3 months | FN | Pet Shop | 2 |  |
| Chocolate | 1.1 | <2 months | M | Breeder | 1 |  |
| Yellow | 10.0 | 6-12 months | MN | Shelter | 1 |  |
| Yellow | 4.5 | 2-3 months | FN | Breeder | 1 |  |
| Chocolate | 6.6 | <2 months | MN | Breeder | 1 |  |
| Yellow | 14.1 | 2-3 months | FN | Pet Shop | 1 |  |
| Yellow | 2.0 | 2-3 months | FN | Breeder | 2 |  |
| Chocolate | 5.5 | <2 months | MN | Breeder | 2 |  |
| Chocolate | 1.3 | 3-6 months | MN | Breeder | 2 |  |
| Black | 8.4 | <2 months | F | Breeder | 3 |  |
| Chocolate | 11.3 | 1-2 years | FN | Breeder | 3 |  |
| Yellow | 4.6 | 2-3 months | MN | Breeder | 1 |  |
| Yellow | 1.4 | <2 months | FN | Breeder | 2 |  |
| Black | 7.0 | 2-3 months | FN | Breeder | 2 | Yes |
| Yellow | 2.9 | <2 months | M | Breeder | 2 |  |
| Yellow | 9.1 | 2-6 years | M | Friend | 1 |  |
| Yellow | 7.5 | 2-3 months | FN | Pet Shop | 2 | Yes |
| Yellow | 0.8 | <2 months | FN | Breeder | 1 | Yes |
| Black | 2.2 | 1-2 years | MN |  | 1 |  |
| Chocolate | 6.6 | 2-6 years | FN | Breeder | 1 |  |
| Yellow | 5.8 | 2-3 months | MN | Breeder | 3 |  |
| Black | 5.8 | 2-3 months | MN |  | 3 |  |
| Yellow | 3.0 | 2-3 months | MN | Breeder | 1 |  |
| Chocolate | 5.1 | 2-3 months | M | Breeder | 2 |  |
| Chocolate | 11.4 | 1-2 years | FN | Breeder | 2 |  |
| Black | 3.5 | 2-6 years | MN | Shelter | 1 | Yes |
| Chocolate | 0.8 | <2 months | F |  |  |  |
| Yellow | 3.7 | 2-3 months | F | Breeder |  |  |
| Black | 3.6 | <2 months | FN | Breeder | 3 |  |
| Yellow | 4.7 | 2-3 months | FN | Breeder | 2 |  |
| Black | 3.0 | 3-6 months | FN | Pet Shop | 2 | Yes |
| Black | 1.9 | 2-3 months | MN | Pet Shop | 2 |  |
| Black | 9.3 | 2-3 months | FN | Breeder | 2 |  |
| Yellow | 9.5 | 2-3 months | FN | Breeder | 2 |  |
| Black | 2.5 | 1-2 years | MN | Shelter | 2 |  |
| Yellow | 5.2 | 6-12 months | MN | Shelter | 1 | Yes |
| Black | 7.8 | 3-6 months | FN | Breeder | 1 | Yes |
| Black | 7 | <2 months | M | Breeder | 4+ |  |
| Black | 10.5 | >6 years | M | Shelter | 2 | Yes |
| Black | 8.7 | 6-12 months | FN |  | 2 |  |
| Black | 1.7 | <2 months | MN | Friend | 1 |  |
| Yellow | 9.4 | 2-3 months | FN | Pet Shop | 2 |  |
| Yellow | 10.9 | >6 years | FN | Friend | 1 |  |
| Black | 3.8 | 3-6 months | MN | Breeder | 1 | Yes |
| Black | 3.9 | <2 months | M | Breeder | 1 |  |
| Chocolate | 2.1 | <2 months | MN | Breeder | 1 |  |
| Black | 3.2 | 2-3 months | FN | Breeder | 2 |  |
| Black | 10.1 | >6 years | FN |  | 2 | Yes |
| Yellow | 9.8 | >6 years | MN |  | 2 |  |
| Black | 6.0 | <2 months | M | Breeder | 1 |  |
| Black | 1.8 | 2-3 months | M | Breeder | 2 | Yes |
| Yellow | 2.1 | 2-3 months | F | Breeder | 1 |  |
| Black | 6.0 | 2-6 years | MN | Breeder | 2 | Yes |
| Black | 1.8 | <2 months | M | Breeder | 2 | Yes |
| Yellow | 9.0 | <2 months | M | Breeder | 1 |  |
| Yellow | 5.4 | <2 months | M | Breeder | 2 |  |
| Yellow | 5.4 | <2 months | FN | Breeder | 2 |  |
| Black | 7.0 | 1-2 years | MN | Breeder | 3 | Yes |
| Black | 1.2 | 2-3 months | MN | Breeder | 3 |  |
| Black | 7.6 | <2 months | FN | Breeder | 2 |  |
| Yellow | 6.6 | <2 months | F | Breeder | 2 |  |
| Yellow |  | >6 years | FN | Shelter | 2 |  |
| Yellow | 5.2 | 2-6 years | M | Assistance Dogs | 2 |  |
| Black | 5.5 | <2 months | MN | Breeder | 4+ |  |
| Black | 8.7 | 2-6 years | FN | Assistance Dogs | 1 | Yes |
| Yellow | 5.8 | 2-6 years | MN | Assistance Dogs | 1 |  |
| Black | 4.2 | 2-6 years | MN | Assistance Dogs | 1 |  |
| Black | 7.3 | 2-6 years | F | Assistance Dogs | 2 | Yes |
| Yellow | 6.0 | 2-6 years | FN | Assistance Dogs | 1 |  |
| Yellow | 3.7 | <2 months | FN | Breeder | 2 | Yes |
| Black | 3.0 | 2-6 years | MN | Assistance Dogs | 1 | Yes |
| Black | 6.4 | <2 months | FN | Breeder | 2 |  |
| Yellow |  | 1-2 years | F | Shelter | 3 |  |
| Chocolate |  | 1-2 years | FN | Shelter | 3 |  |
| Yellow | 10.9 | <2 months | FN |  | 2 |  |
| Black | 9.6 | <2 months | FN | Breeder | 2 |  |
| Chocolate | 1.9 | <2 months | M | Breeder | 4+ | Yes |
| Chocolate | 1.0 | <2 months | FN | Breeder | 1 |  |
| Chocolate | 1.9 | <2 months | FN | Breeder | 2 |  |
| Yellow | 1.0 | 6-12 months | MN | Shelter | 2 |  |
| Black | 5.1 | 2-3 months | FN | Breeder | 2 | Yes |
| Yellow | 3.1 | 2-6 years | FN |  | 2 |  |
| Yellow | 11.9 | 2-6 years | F | Assistance Dogs | 1 |  |
| Yellow | 6.5 | <2 months | F | Breeder | 1 |  |
| Black | 9.3 | 2-3 months | FN | Breeder | 1 | Yes |
| Black | 10.5 | >6 years | FN | Friend | 1 |  |
| Yellow | 12.2 | 2-3 months | FN | Breeder | 2 |  |
| Yellow | 6.2 | 2-3 months | FN | Breeder | 2 |  |
| Yellow | 3.6 | 2-3 months | MN | Breeder | 1 |  |
|  |  |  |  |  |  |  |
