# Supplementary material for: Association between coat colour and the behaviour of Australian Labrador retrievers
Source: Canine Genet Epidemiol. 2019 Nov 30;6:10. doi: 10.1186/s40575-019-0078-z (PMC6884874; doi:10.1186/s40575-019-0078-z)
Supplement: Supplementary file 2 — Additional file 2: Table S2. Australian Canine Behaviour Survey behavioural traits, calculating scores and their definitions. (DOCX 16 kb) [file 40575_2019_78_MOESM2_ESM.docx]

Table S2: Australian Canine Behaviour Survey behavioural traits, calculating scores, and their definitions. Definitions based on C-BARQ ([Hsu and Serpell, 2003](#_ENREF_18); [Duffy et al., 2008](#_ENREF_8))

| **Behaviour factor** | **Items / No. items** | **Definition** |
| --- | --- | --- |
| Agitated when ignored | 73+74 / 2 | Dog becomes agitated when you show affection for another person or animal |
| Attachment/Attention-seeking behaviour | 69+70+71+72+73+74 /6 | Dog maintains close proximity to owners or household members, solicits attention, becomes agitated when the owner gives attention to a third party |
| Barking | 98 | Dog barks persistently when alarmed or excited |
| Chasing behaviour | 27+75+76+77 / 4 | Dog pursues cats, birds and other small animals, given the opportunity |
| Coprophagia | 80 | Dog eats own or other animals’ faeces |
| Dog-directed aggression | 23+24+26+29 / 4 | Dog shows aggressive responses when approached by unfamiliar dogs |
| Dog-directed fear | 45+46+53+54 / 4 | Dog shows fearful responses when approached by unfamiliar dogs |
| Energy levels | 92+93 / 2 | Dog shows highly energetic and playful behaviour |
| Excitability | 63+64+65+66+67+68 / 6 | Dog shows strong reaction to potentially exciting or arousing events such as going for a walk or car trip, doorbells, arrival of visitors, owner arriving home |
| Familiar dog aggression | 32+33+34+35 / 4 | Dog shows aggressive responses towards other dog(s) in household, competing for resources |
| Licking behaviour | 99+100 / 2 | Dog licks own body, people or objects excessively |
| Mounting behaviour | 82 | Dog mounts objects, furniture or people |
| Noise fear | 38+44 / 2 | Dog shows fearful or wary responses to sudden or loud noises, thunderstorms, fireworks |
| Non-social fear | 38+41+42+44+47+48 / 6 | Dog shows fearful or wary responses to sudden or loud noises, thunderstorms, traffic, unfamiliar objects and situations |
| Owner-directed aggression | 9+13+14+17+19+25+30+31 / 8 | Dog shows aggressive responses to the owner or other household member when verbally corrected, challenged, stepped over, handled, or when approached while in possession of food or objects |
| Separation-related behaviour | 55+56+57+58+59+60+61+62 / 8 | Dog vocalises, engages in destructive behaviour, or exhibits signs of anxiety when left, or about to be left, alone |
| Stranger-directed aggression | 10+11+12+15+16+18+20+21+22+28 / 10 | Dog shows aggressive responses to strangers approaching or invading the dog's territory |
| Stranger-directed fear | 36+37+39+10 / 4 | Dog shows fearful or wary responses when approached by unfamiliar people |
| Touch sensitivity | 43+49+50+52 / 4 | Dog shows fearful or wary response to handling procedures such as bathing, grooming, nail clipping and veterinary examination |
| Trainability | 1+2+3+4+5+6+7+8 / 8 | Dog shows willingness to attend and obey owner, an ability to learn new tasks, fetch objects, positively responds to correction, and ignores distractions |
| Unusual behaviours | 94+95+96+97+99+100+101 / 7 | Dog stares at nothing, snaps at invisible flies, chases tail, chases shadows, licks excessively or displays repetitive, bizarre behaviour |

Scoring: Never = 0, Seldom = 1, Sometimes = 2, Often = 3, Always = 4 except for items 5, 6 & 7 where scores are reversed, e.g. Never = 4 to Always = 0
