# Supplementary material for: Association between coat colour and the behaviour of Australian Labrador retrievers
Source: Canine Genet Epidemiol. 2019 Nov 30;6:10. doi: 10.1186/s40575-019-0078-z (PMC6884874; doi:10.1186/s40575-019-0078-z)
Supplement: Supplementary file 3 — Additional file 3: Australian Canine Behaviour Survey. [file 40575_2019_78_MOESM3_ESM.docx]

Name: ……………………………………

Phone: ……………………………………

Email: ……………………………………

**AUSTRALIAN DOG BEHAVIOUR SURVEY**

| Thank you for taking the time to complete this questionnaire. It will remain confidential.  First we need some information about your dog and household.  Could you please answer the following, circling the most appropriate response where needed. | | | | | | | |
| --- | --- | --- | --- | --- | --- | --- | --- |
| Dog’s Name: | | | | | | | |
| Breed: Labrador Retriever Golden Retriever | | | | | | | |
| Colour: Yellow Chocolate Black Gold | | | | | | | |
| Sex: Male Male Neuter Female Female Neuter | | | | | | | |
| Age: yrs mths | | Age Acquired: yrs mths | | | | | |
| Acquired from: Breeder Shelter Pet Shop Friend Guide Dogs Customs Other  NB. If you bred dog yourself, please circle ‘Breeder’ | | | | | | | |
| Number of dogs in household: 1 2 3 4 5+ | | | | | | | |
| Main carer of dog: Myself Partner Other | | | | | | | |
|  | | | | | | | |
| **Section 1: Training and obedience** | | | | | | | |
| Some dogs are more obedient and trainable than others. By ticking the appropriate choices, please indicate how trainable or obedient your dog has been in each of the following situations in the recent past. | | | | | | | |
|  | 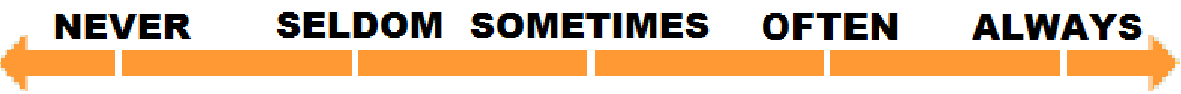 | | | | | | 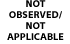 |
| **1.** When off the leash, returns immediately when called. |  | |  |  |  |  |  |
| **2.** Obeys the "sit" command immediately. |  | |  |  |  |  |  |
| **3.** Obeys the "stay" command immediately. |  | |  |  |  |  |  |
| **4.** Seems to attend/listen closely to everything you say or do. |  | |  |  |  |  |  |
| **5.** Slow to respond to correction or punishment; "thick-skinned". |  | |  |  |  |  |  |
| **6.** Slow to learn new tricks or tasks. |  | |  |  |  |  |  |
| **7.** Easily distracted by interesting sights, sounds, or smells. |  | |  |  |  |  |  |
| **8.** Will "fetch" or attempt to fetch sticks, balls, or objects. |  | |  |  |  |  |  |
|  | | | | | | | |
| **Section 2: Aggression** | | | | | | | |
| Some dogs display aggressive behaviour from time to time. Typical signs of moderate aggression in dogs include growling and baring teeth. More serious aggression generally includes snapping, lunging, biting, or attempting to bite. | | | | | | | |
| By ticking the following scales, please indicate your own dog's recent tendency to display aggressive behaviour in each of the following contexts: | | | | | | | |
|  | 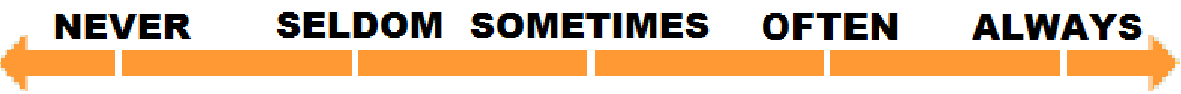 | | | | | | 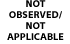 |
| **9.** When verbally corrected or punished (scolded, shouted at, etc.) by you or a household member. |  | |  |  |  |  |  |
| **10.** When approached directly by an unfamiliar **adult** while being walked/exercised on a leash. |  | |  |  |  |  |  |
| **11.** When approached directly by an unfamiliar **child** while being walked/exercised on a leash. |  | |  |  |  |  |  |
| **12.** Toward unfamiliar persons approaching the dog while s/he is in your car (at the service station, for example). |  | |  |  |  |  |  |
| **13.** When toys, bones or other objects are taken away by a household member. |  | |  |  |  |  |  |
| **14.** When bathed or groomed by a household member. |  | |  |  |  |  |  |
| **15.** When an unfamiliar person approaches you or another member of your family at home. |  | |  |  |  |  |  |
| **16.** When unfamiliar persons approach you or another member of your family away from your home. |  | |  |  |  |  |  |
| **17.** When approached directly by a household member while s/he is eating. |  | |  |  |  |  |  |
| **18.** When mailmen or other delivery workers approach your home. |  | |  |  |  |  |  |
| **19.** When his/her food is taken away by a household member. |  | |  |  |  |  |  |
| **20.** When strangers walk past your home while your dog is outside or in the yard. |  | |  |  |  |  |  |
| **21.** When an unfamiliar person tries to touch or pet the dog. |  | |  |  |  |  |  |
| **22.** When joggers, cyclists, rollerbladers or skateboarders pass your home while your dog is outside or in the yard. |  | |  |  |  |  |  |
| **23.** When approached directly by an unfamiliar **male** dog while being walked/exercised on a leash. |  | |  |  |  |  |  |
| **24.** When approached directly by an unfamiliar **female** dog while being walked/exercised on a leash. |  | |  |  |  |  |  |
| **25.** When stared at directly by a member of the household. |  | |  |  |  |  |  |
| **26.** Toward unfamiliar dogs visiting your home. |  | |  |  |  |  |  |
| **27.** Toward cats or other small animals entering your yard. |  | |  |  |  |  |  |
| **28.** Toward unfamiliar persons visiting your home. |  | |  |  |  |  |  |
| **29.** When barked, growled, or lunged at by another (unfamiliar) dog. |  | |  |  |  |  |  |
| **30.** When stepped over by a member of the household. |  | |  |  |  |  |  |
|  | 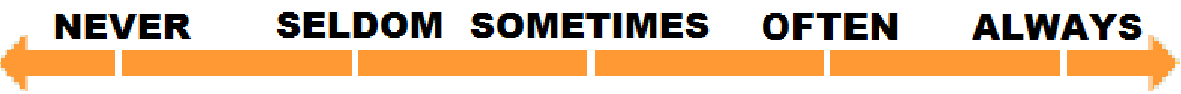 | | | | | | 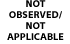 |
| **31.** When you or a household member retrieves food or objects stolen by the dog. |  | |  |  |  |  |  |
| **32.** Towards another (familiar) dog in your household. |  | |  |  |  |  |  |
| **33.** When approached at a favourite resting/sleeping place by another (familiar) household dog. |  | |  |  |  |  |  |
| **34.** When approached while eating by another (familiar) household dog. |  | |  |  |  |  |  |
| **35.** When approached while playing with/chewing a favourite toy, bone, object, etc., by another (familiar) household dog. |  | |  |  |  |  |  |
| Are there any other situations in which your dog is sometimes aggressive? If so, please describe briefly: |  | | | | | | |
|  | | | | | | | |
| **Section 3: Fear and anxiety** | | | | | | | |
| Dogs sometimes show signs of anxiety or fear when exposed to particular sounds, objects, persons or situations. Typical signs of mild to moderate fear include: avoiding eye contact, avoidance of the feared object, crouching or cringing with tail lowered or tucked between the legs, whimpering and whining, freezing, and shaking and trembling. Extreme fear is characterized by exaggerated cowering, and/or vigorous attempts to escape, retreat or hide from the feared object, person or situation. | | | | | | | |
| Please indicate your own dog's recent tendency to display fearful behaviour in each of the following contexts: | | | | | | | |
|  | 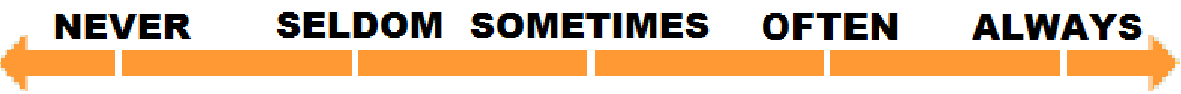 | | | | | | 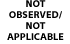 |
| **36.** When approached directly by an unfamiliar **adult** while away from your home. |  | |  |  |  |  |  |
| **37.** When approached directly by an unfamiliar **child** while away from your home. |  | |  |  |  |  |  |
| **38.** In response to sudden or loud noises (e.g. vacuum cleaner, car backfire, road drills, objects being dropped, etc.). |  | |  |  |  |  |  |
| **39.** When unfamiliar persons visit your home. |  | |  |  |  |  |  |
| **40.** When an unfamiliar person tries to touch or pet the dog. |  | |  |  |  |  |  |
| **41.** In heavy traffic. |  | |  |  |  |  |  |
| **42.** In response to strange or unfamiliar objects on or near the sidewalk (e.g. plastic bags, leaves, litter, flags flapping, etc.). |  | |  |  |  |  |  |
| **43.** When examined/treated by a veterinarian. |  | |  |  |  |  |  |
| **44.** During thunderstorms, firework displays, or similar events. |  | |  |  |  |  |  |
| **45.** When approached directly by an unfamiliar dog of the same or larger size. |  | |  |  |  |  |  |
| **46.** When approached directly by an unfamiliar dog of smaller size. |  | |  |  |  |  |  |
| **47.** When first exposed to unfamiliar situations (e.g. first car trip, first time in elevator, first visit to veterinarian, etc.). |  | |  |  |  |  |  |
|  | 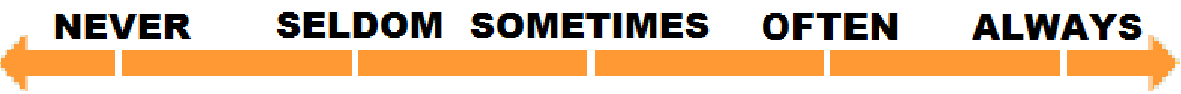 | | | | | | 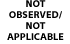 |
| **48.** In response to wind or wind-blown objects. |  | |  |  |  |  |  |
| **49.** When having nails clipped by a household member. |  | |  |  |  |  |  |
| **50.** When groomed or bathed by a household member. |  | |  |  |  |  |  |
| **51.** When stepped over by a member of the household. |  | |  |  |  |  |  |
| **52.** When having his/her feet towelled by a member of the household. |  | |  |  |  |  |  |
| **53.** When unfamiliar dogs visit your home. |  | |  |  |  |  |  |
| **54.** When barked, growled, or lunged at by an unfamiliar dog. |  | |  |  |  |  |  |
|  | | | | | | | |
| **Section 4: Separation-related behaviour** | | | | | | | |
| Some dogs show signs of anxiety or abnormal behaviour when left alone, even for relatively short periods of time. Thinking back over the recent past, how often has your dog shown each of the following signs of separation-related behaviour when left, or about to be left, on its own: | | | | | | | |
|  | 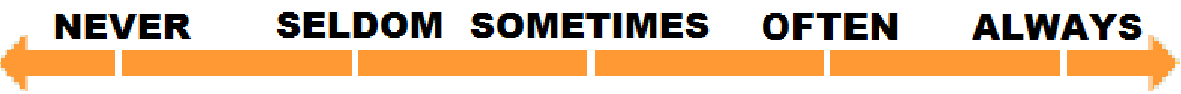 | | | | | | 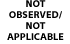 |
| **55.** Shaking, shivering, or trembling. |  | |  |  |  |  |  |
| **56.** Excessive salivation. |  | |  |  |  |  |  |
| **57.** Restlessness, agitation, or pacing. |  | |  |  |  |  |  |
| **58.** Whining. |  | |  |  |  |  |  |
| **59.** Barking. |  | |  |  |  |  |  |
| **60.** Howling. |  | |  |  |  |  |  |
| **61.** Chewing or scratching at doors, floor, windows, curtains, etc. |  | |  |  |  |  |  |
| **62.** Loss of appetite. |  | |  |  |  |  |  |
| Are there any other situations in which your dog is fearful or anxious? If so, please describe briefly: |  | | | | | | |
|  | | | | | | | |
| **Section 5: Excitability** | | | | | | | |
| Some dogs show relatively little reaction to sudden or potentially exciting events and disturbances in their environment, while others become highly excited at the slightest novelty. Signs of mild to moderate excitability include increased alertness, movement toward the source of novelty, and brief episodes of barking. Extreme excitability is characterized by a general tendency to over-react. The excitable dog barks or yelps hysterically at the slightest disturbance, rushes toward and around any source of excitement, and is difficult to calm down. | | | | | | | |
| Please indicate your own dog's recent tendency to show extreme excitability in each of the following contexts: | | | | | | | |
|  | 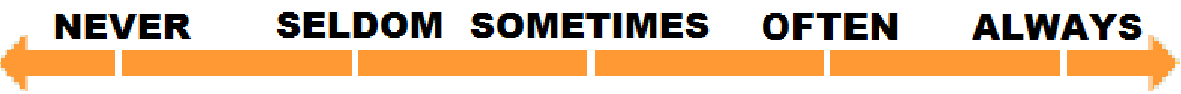 | | | | | | 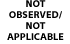 |
| **63.** When you or other members of the household come home after a brief absence. |  | |  |  |  |  |  |
|  | 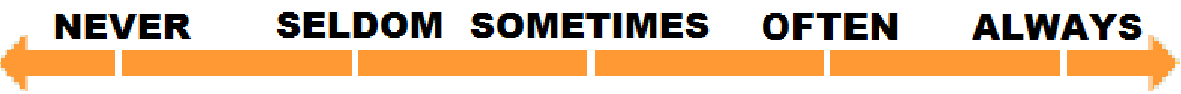 | | | | | | 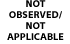 |
| **64.** When playing with you or other members of your household. |  | |  |  |  |  |  |
| **65.** When doorbell rings. |  | |  |  |  |  |  |
| **66.** Just before being taken for a walk. |  | |  |  |  |  |  |
| **67.** Just before being taken on a car trip. |  | |  |  |  |  |  |
| **68.** When visitors arrive at your home. |  | |  |  |  |  |  |
| Are there any other situations in which your dog sometimes becomes over-excited? If so, please describe briefly: |  | | | | | | |
|  | | | | | | | |
| **Section 6: Attachment and attention-seeking** | | | | | | | |
| Most dogs are strongly attached to their people, and some demand a great deal of attention and affection from them. Thinking back over the recent past, how often has your dog shown each of the following signs of attachment or attention-seeking: | | | | | | | |
|  | 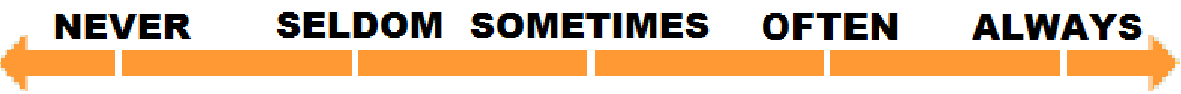 | | | | | | 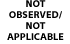 |
| **69.** Displays a strong attachment for one particular member of the household. |  | |  |  |  |  |  |
| **70.** Tends to follow you (or other members of the household) about the house, from room to room. |  | |  |  |  |  |  |
| **71.** Tends to sit close to, or in contact with, you (or others) when you are sitting down. |  | |  |  |  |  |  |
| **72.** Tends to nudge, nuzzle or paw you (or others) for attention when you are sitting down. |  | |  |  |  |  |  |
| **73.** Becomes agitated (whines, jumps up, tries to intervene) when you (or others) show affection for another person. |  | |  |  |  |  |  |
| **74.** Becomes agitated (whines, jumps up, tries to intervene) when you (or others) show affection for another dog or animal. |  | |  |  |  |  |  |
|  | | | | | | | |
| **Section 7: Miscellaneous** | | | | | | | |
| Dogs display a wide range of miscellaneous behaviour problems in addition to those already covered by this questionnaire. Thinking back over the recent past, please indicate how often your dog has shown any of the following behaviours: | | | | | | | |
|  | 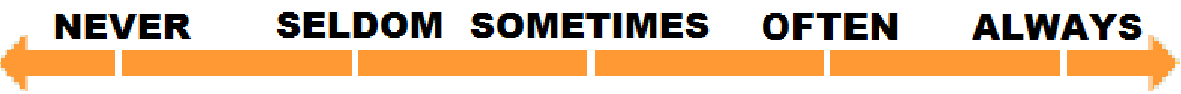 | | | | | | 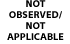 |
| **75.** Chases or would chase cats given the opportunity. |  | |  |  |  |  |  |
| **76.** Chases or would chase birds given the opportunity. |  | |  |  |  |  |  |
| **77.** Chases or would chase rabbits and other small animals given the opportunity. |  | |  |  |  |  |  |
| **78.** Escapes or would escape from home or yard given the chance. |  | |  |  |  |  |  |
| **79.** Rolls in animal droppings or other "smelly" substances. |  | |  |  |  |  |  |
|  | 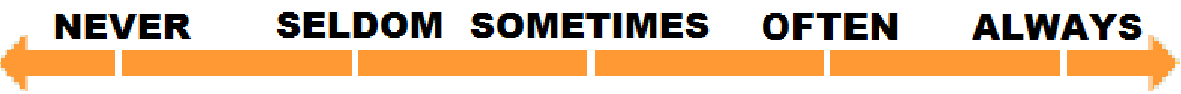 | | | | | | 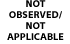 |
| **80.** Eats own or other animals' droppings or faeces. |  | |  |  |  |  |  |
| **81.** Chews inappropriate objects. |  | |  |  |  |  |  |
| **82.** "Mounts" objects, furniture, or people. |  | |  |  |  |  |  |
| **83.** Begs persistently for food when people are eating. |  | |  |  |  |  |  |
| **84.** Steals food. |  | |  |  |  |  |  |
| **85.** Nervous or frightened on stairs. |  | |  |  |  |  |  |
| **86.** Pulls excessively hard when on the leash. |  | |  |  |  |  |  |
| **87.** Urinates against objects/furnishings in your home. |  | |  |  |  |  |  |
| **88.** Urinates when approached, petted, handled or picked up. |  | |  |  |  |  |  |
| **89.** Urinates when left alone at night, or during the daytime. |  | |  |  |  |  |  |
| **90.** Defecates when left alone at night, or during the daytime. |  | |  |  |  |  |  |
| **91.** Hyperactive, restless, has trouble settling down. |  | |  |  |  |  |  |
| **92.** Playful, puppyish, boisterous. |  | |  |  |  |  |  |
| **93.** Active, energetic, always on the go. |  | |  |  |  |  |  |
| **94.** Stares intently at nothing visible. |  | |  |  |  |  |  |
| **95.** Snaps at invisible flies. |  | |  |  |  |  |  |
| **96.** Chases own tail/hind end. |  | |  |  |  |  |  |
| **97.** Chases/follows shadows, light spots, etc. |  | |  |  |  |  |  |
| **98.** Barks persistently when alarmed or excited. |  | |  |  |  |  |  |
| **99.** Licks him/herself excessively. |  | |  |  |  |  |  |
| **100.** Licks people or objects excessively. |  | |  |  |  |  |  |
| **101.** Displays other bizarre, strange, or repetitive behaviour(s)*. |  | |  |  |  |  |  |
| *Please describe briefly: |  | | | | | | |

This survey is based largely on the Canine Behavior Assessment & Research Questionnaire (CBARQ)
developed by Dr. James Serpell at Pennsylvania State University.
